# Electronically Stabilized Copoly(Styrene-Acrylic Acid) Submicrocapsules Prepared by Miniemulsion Copolymerization

**DOI:** 10.3390/polym9070291

**Published:** 2017-07-20

**Authors:** Minkwan Kim, Yura Hwang, Han Do Ghim

**Affiliations:** Department of Textile System Engineering, Kyungpook National University, 80 Daehakro, Bukgu, Daegu 41566, Korea; gundamzxv@naver.com (M.K.); youra3670@naver.com (Y.H.)

**Keywords:** styrene, acrylic acid, submicrocapsule, miniemulsion copolymerization, zeta potential

## Abstract

This work reports the preparation and characterization of poly(styrene-acrylic acid) (St/AA) submicrocapsules by using the miniemulsion copolymerization method. AA was introduced to miniemulsion polymerization of St to increase the zeta potential and the resulting electrostatic stability of St/AA submicrocapsules. Phytoncide oil was adopted as the core model material. Miniemulsion copolymerization of St and AA was conducted at a fixed monomer concentration (0.172 mol) with a varying monomer feed ratio [AA]/[St] (0.2, 0.25, 0.33, 0.5, and 1.0). Concentrations of initiator (azobisisobutyronitrile; 1.0 × 10^−3^, 2.0 × 10^−3^, 3.0 × 10^−3^, and 4.0 × 10^−3^ mol/mol of monomer) and surfactant (sodium dodecyl sulfate; 0.6 × 10^−3^, 1.0 × 10^−3^, and 1.4 × 10^−3^ mol) were also controlled to optimize the miniemulsion copolymerization of St and AA. Dynamic light scattering and microscopic analyses confirmed the optimum condition of miniemulsion copolymerization of St and AA. Long-term colloidal stability of aqueous St/AA submicrocapsule suspension was evaluated by using Turbiscan^TM^ Lab. In this work, the optimum condition for miniemulsion copolymerization of St and AA was determined ([AA]/[St] = 0.33; [SDS] = 1.0 × 10^−3^ mol; [AIBN] = 2.0 × 10^−3^ mol/mol of monomer). St/AA submicrocapsules prepared at the optimum condition (392.6 nm and −55.2 mV of mean particle size and zeta potential, respectively) showed almost no variations in backscattering intensity (stable colloids without aggregation).

## 1. Introduction

Deliveries of functional hydrophobic materials using polymeric nanoparticles have been of great interest in biomedical sciences [1,2,3,4,5,6]. Furthermore, emulsions containing polymers can be applied to modify the surface of porous materials, endowing hydrophobic characteristics [7,8,9].

There are many methodologies for preparing nanoparticles including miniemulsion polymerization [10,11,12,13], interfacial polymerization, solvent evaporation, solvent deposition, nanoprecipitation, desolvation of natural polymers [14], and emulsification–diffusion [15,16,17]. Among these methods, miniemulsion polymerization is conducted by adopting cosurfactants such as hexadecane, cetyl alcohol, and so on. The existence of surfactant and cosurfactant ensures the formation of stable o/w miniemulsion (or nanoemulsion) under high shear without coalescence and Ostwald ripening [18]. Unfortunately, however, resulting nanoparticles and submicroparticles show insufficient dispersibility because of particle coagulation due to a lack of surface charge.

Zeta potential is the potential difference between the dispersion medium (water, in this case) and the stationary layer of fluid attached to the dispersed particle [19]. Therefore, the zeta potential can be used as a key indicator of the stability of colloidal dispersions because the magnitude of the zeta potential indicates the degree of electrostatic repulsion between particles in a dispersion. For small particles, high zeta potential will confirm the electrostatic stability, preventing aggregation in the dispersion. If the zeta potential is small, this electrostatic repulsion may not be implemented by exceeding attractive forces, resulting in the flocculation of particles. Mandzy et al. [20] reported that reagglomeration of the TiO_2_ nanoparticle dispersion could be prevented by electrostatic stabilization when its zeta potential value was less than −30 mV or greater than +30 mV. Many other researchers have investigated the effects of zeta potential on particle stability for metallic powders including zirconia and titania [21,22,23,24].

Polystyrene nanocapsules have been adopted as vehicles for photochromophores [25], phase change materials [26], and so on, by virtue of their ease of preparation and thermal stability. However, chronic particle aggregation of polystyrene nanoparticles has constrained their practical usage. Even though several researchers have investigated the miniemulsion polymerization of styrene (St) and acrylic acid (AA) to enhance the colloidal stability of polystyrene nanocapsules, copolymerization of AA and St with over 20 wt % of AA has rarely been considered for water compatibility of AA [27,28,29]. In this work, we adopted AA to miniemulsion polymerization of St to increase the zeta potential and resulting electrostatic stability of St/AA submicrocapsules containing phytoncide oil. To this aim, St/AA submicrocapsules were prepared at over 20 wt % of AA content. Mean particle size and zeta potential of St/AA submicrocapsules were then analyzed by dynamic light scattering (DSL). Dispersability of submicrocapsules was also evaluated by Turbiscan^TM^ Lab to confirm the sink stability over time.

## 2. Materials and Methods

### 2.1. Materials

St and AA were purchased from Aldrich Chemical (St. Louis, MI, USA) and used as monomers after distillation. Azobisisobutyronitrile (AIBN) and divinyl benzene (DVB) were also provided by Aldrich Chemical (St. Louis, MI, USA) and used as initiator and crosslinking agent, respectively. Dae Jung Chemical (Siheung, Korea) supplied sodium dodecyl sulfate (SDS), which is used as surfactant. *n*-Hexadecane was purchased from Alfa Aesar (Ward Hill, MA, USA) and used as cosurfactant. Distilled water was of Milli-Q quality (Millipore, Billerica, MA, USA). Phytoncide oil was provided by CNG Co. (Daegu, Korea). All the reagents were of either HPLC grade or American Chemical Society analytical grade.

### 2.2. Miniemulsion Copolymerization

SDS (0.6~1.4 × 10^−3^ mol) was dissolved in distilled water to prepare the water phase. St and AA were homogeneously mixed with phytoncide oil, and *n*-hexadecane to form the oil phase. These solutions were emulsified with homogenizer for 10 min at 19,000 rpm to prepare micelles in submicrometer range. AIBN (initiator, 1.0~4.0 × 10^−3^ mol/mol of monomer) was added at 60 °C and copolymerization was maintained for 6 h. The pH of the reaction system was slightly lowered with the increase of the amount of AA, ranging from 2.3 to 2.6. Detailed experimental conditions are presented in Table 1.

### 2.3. Characterization

The size and morphology of St/AA submicrocapsules were analyzed by scanning electron microscopy (SEM) (SU8220, Hitachi, Tokyo, Japan). The mean particle diameters and zeta potentials of St/AA submicrocapsules were also determined using DLS (ELS8000, Photal Otsuka Electronics, Osaka, Japan) equipped with vertically polarized light supplied by a He-Ne laser, operated at 10 mW. DLS measurements were performed at room temperature. The dispersion stability of St/AA submicroparticles in the aqueous phase was evaluated from the time variation of backscattered light by using the Turbiscan^TM^ Lab (Formulaction LAB, Toulouse, France). All the measurements were repeated three times.

## 3. Results and Discussion

### 3.1. Effects of Monomer Feed Ratio

The aim of miniemulsion copolymerization of St and AA is to increase the stability and prevent the coagulation of submicrocapsules containing functional materials in the core (phytoncide oil, in this case) by virtue of the enhanced zeta potential of submicrocapsules due to –COO^−^ functional groups of AA. The salient feature of our current study is that miniemulsion copolymerization of St with AA yields electronically stabilized submicrocapsules which can be used as carriers for the lypophilic core.

**Figure 1:**
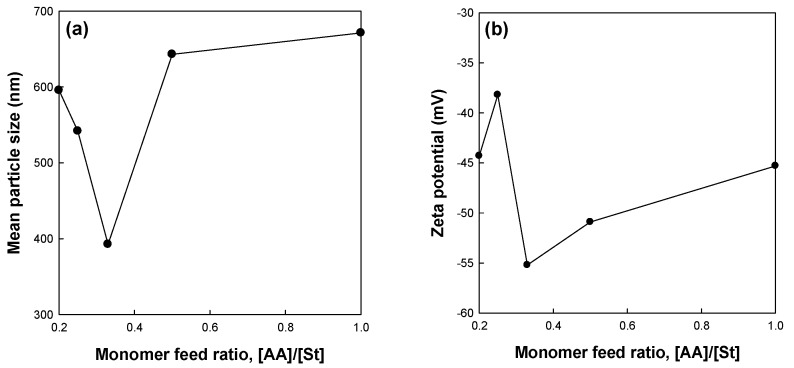
Effects of the monomer feed ratio on (**a**) mean particle size and (**b**) zeta potential of the St/AA submicroparticles prepared at 1.0 × 10^−3^ mol and 2.0 × 10^−3^ mol/mol of monomer for SDS and AIBN concentrations, respectively.

**Figure 2:**
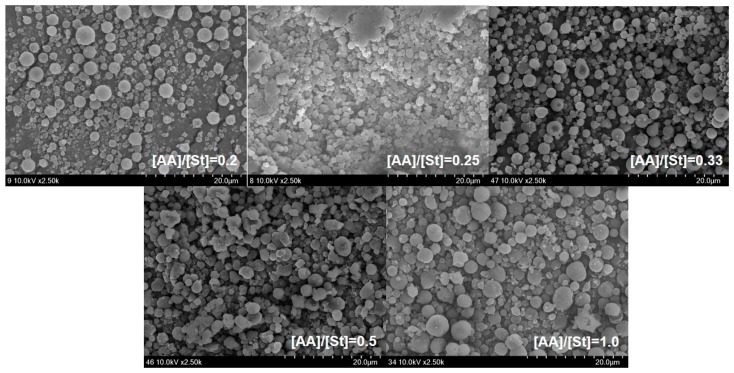
SEM photographs of St/AA submicrocapsules prepared at 1.0 × 10^−3^ mol and 2.0 × 10^−3^ mol/mol of monomer for SDS and AIBN concentrations, respectively, with a varying monomer feed ratio.

Figure 1 shows the effects of the monomer feed ratio [AA]/[St] of miniemulsion copolymerization of St and AA on the mean particle size and zeta potential of the St/AA submicrocapsule. The particle size of the St/AA submicroparticle presents a minimum value of 392.6 nm at 0.33 of the monomer feed ratio (Figure 1a). The particle size of the St/AA submicroparticles decreased with the increase of [AA] at [AA]/[St] = 0.2~0.33 range, which was ascribed to the increased surface potential of micelles. For increased [AA]/[St] over 0.33, however, there was a steep increase in the mean particle size of the submicroparticles. As is well known, copolymerization behavior is mainly affected by the different reactivities of monomers. Compared with St, AA polymerizes vary rapidly, especially at low pH. Therefore, the increase of AA led inevitably to longer polyacrylic blocks in the copolymer formation at the particle–water interface, and increased the particle diameter. The increase in particle size with the increase in the amount of AA results in the formation of a hydrophilic swollen layer around the particle [27]. Absolute value of zeta potential (Figure 1b) increased to −55.2 mV with the increases of [AA] at [AA]/[St] = 0.2~0.33 range. The decrease in the absolute zeta potential value at over [AA]/[St] = 0.33 was attributed to the particle agglomeration in the aqueous phase due to the increased hydrophilic swollen layer of the particles and resulting reduction in surface area [29,30]. This can also be confirmed from SEM photographs presented in Figure 2. The St/AA submicroparticles prepared at [AA]/[St] = 0.5 and 1.0 showed crumpled and contorted morphology, which arose from the agglomeration of particles due to the fusion of hydrophilic swollen layers in dispersive medium. Fused morphology of St/AA submicrocapsules indicates the low thermal durability of the wall of submicrocapsules that is unable to tolerate the deposition process prior to microscopic analysis.

### 3.2. Effects of Surfactant Concentration

**Figure 3:**
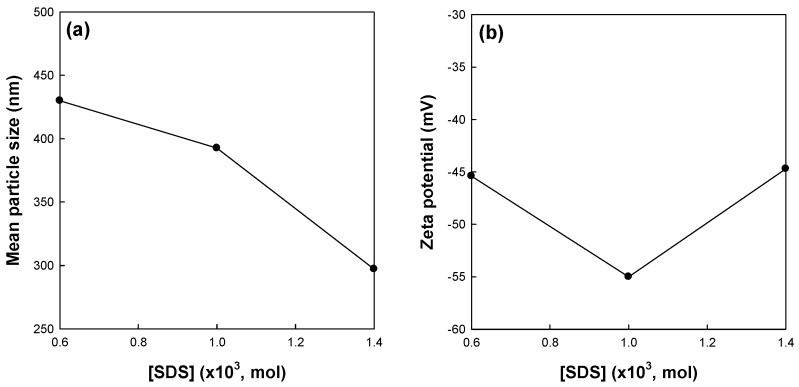
Effects of surfactant concentration on (**a**) mean particle size and (**b**) zeta potential of St/AA submicroparticles prepared at 0.33 and 2.0 × 10^−3^ mol/mol of monomer for [AA]/[St] and AIBN concentration, respectively.

Figure 3 shows the effects of surfactant concentration on the mean particle size and zeta potential of St/AA submicrocapsules prepared at 0.33 and 2.0 × 10^−3^ mol/mol of monomer for [AA]/[St] and AIBN concentrations, respectively. The mean particle size of the submicrocapsules decreased with the increasing surfactant concentration (Figure 3a). This result is in good agreement with those of other researchers, suggesting that more SDS molecules are available for stabilizing the oil–water interfacial area generated during homogenization at a higher level of SDS [29,31,32,33]. The zeta potential of submicrocapsules, however, showed the optimum value at 1.0 × 10^−3^ mole of surfactant concentration (Figure 3b).

**Figure 4:**
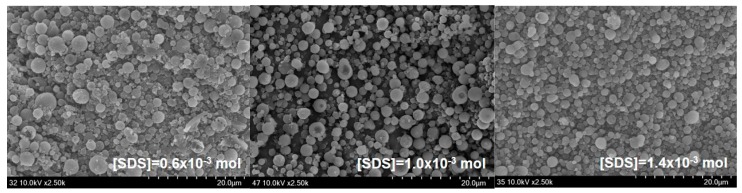
SEM photographs of St/AA submicrocapsules prepared at 0.33 and 2.0 × 10^−3^ mol/mol of monomer for [AA]/[St] and AIBN concentration, respectively, with varying surfactant content.

Elimelech and O’Melia [34] reported that the collision efficiency of colloidal particles is independent of particle size. They also suggested that coupling of electrodynamics and hydrodynamics, and the possible effects of surface roughness have a significant effect on the kinetics of particle–particle interaction. With the increase in the concentration of SDS, a large number of smaller micelles can be obtained. Therefore, St/AA submicrocapsules prepared from these micelles are supposed to have a relatively thin and low molecular weight electrostatic polyacrylic shell. This meager swollen layer will become vulnerable to aggregation and, as a result, show lower zeta potential. This reduction in the absolute value of zeta potential at higher SDS content can be confirmed by Figure 4 which presents SEM photographs of St/AA submicroparticles with varying surfactant concentrations.

### 3.3. Effects of Initiator Concentration

**Figure 5:**
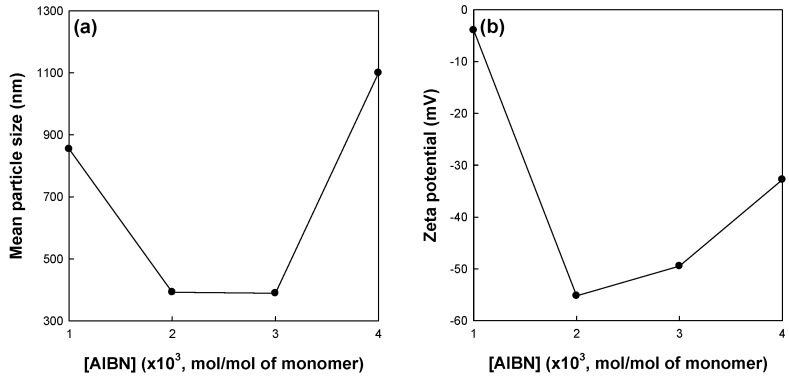
Effects of the initiator concentration on (**a**) mean particle size and (**b**) zeta potential of St/AA submicroparticles prepared at 0.33 and 1.0 × 10^−3^ mol for [AA]/[St] and SDS concentration, respectively.

Figure 5 shows the effects of the initiator concentration on the mean particle size and zeta potential of St/AA submicrocapsules prepared at 0.33 and 1.0 × 10^−3^ mol for [AA]/[St] and SDS concentrations, respectively. As is well known, the initiator concentration greatly affects the reaction rate and extent of radical polymerization. The increase in initiator concentration at a fixed amount of surfactant will increase the possibility and frequency of droplet nucleation in a micelle. At this point, preference of AA for copolymerization with St is no longer maintained. Therefore, the hydrophilic swollen layer composed of longer polyacrylic blocks and the resulting zeta potential of St/AA submicrocapsules were assumed to be diminished for higher initiator concentrations. On the other hand, a limited amount of initiator could not properly activate the droplet nucleation. At an initiator concentration of 1.0 × 10^−3^ mol/mol of monomer, it was proposed that submicrocapsules were formed insufficiently, which was confirmed by large particle size and low zeta potential. As shown in Figure 5b, the St/AA submicrocapsule prepared at 1.0 × 10^−3^ mol/mol of monomer of the initiator concentration shows no surface potential (almost zero potential) which means that immense coagulation of particles will occur.

### 3.4. Colloidal Stability

In this work, the long-term stability of St/AA submicrocapsules containing phytoncide oil was investigated by evaluating both the optical transmission and the photon backscattering profiles of the aqueous dispersion of submicrocapsules by using the Turbiscan^TM^ Lab (Formulaction, Toulouse, France) [35]. Measurements were carried out using a pulsed near infrared LED at a wavelength of 880 nm for 24 h. Two different synchronous optical sensors received the light transmitted through and backscattered by samples at an angle of 180° and 45° with respect to the incident radiation, respectively. Transmitted and backscattered light flux were correlated as a percentage to those of reference standards. If there was no variation greater than 2% in the backscattering profile, it can be considered as a stable formulation [36].

**Figure 6:**
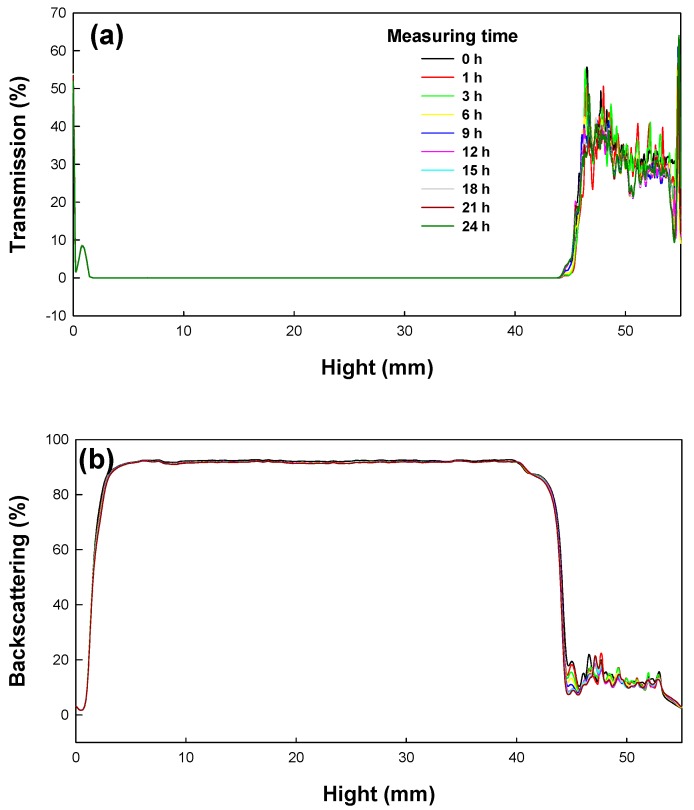
Transmission and backscattering profiles of St/AA submicrocapsule aqueous dispersion. (Prepared at the optimum miniemulsion condition of [AA]/[St] = 0.33, [SDS] = 1.0 × 10^−3^ mol, [AIBN] = 2.0 × 10^−3^ mol/mol of monomer).

The transmission and backscattering profiles of the St/AA submicrocapsule aqueous dispersion prepared at the optimum condition ([AA]/[St] = 0.33; [SDS] = 1.0 × 10^−3^ mol; [AIBN] = 2.0 × 10^−3^ mol/mol of monomer) are shown in Figure 6. Variations of transmission and backscattering profiles were not correlated to the destabilization processes below the sample height of 5 mm and over that of 40 mm, the values having been determined by enclosed air in the bottom and/or on the top of the cylindrical glass tube, respectively. Analysis of the submicrocapsule dispersion showed that backscattering was the prevalent signal and there were almost no variations in the backscattering intensity for the entire test height. From this result, it can be confirmed that St/AA submicrocapsules prepared at the optimum condition could be formulated as stable colloids without aggregation.

## 4. Conclusions

It was shown that St/AA submicrocapsules can be obtained by miniemulsion copolymerization of AA and St with lypophilic core materials. There was an optimum condition for obtaining electronically stable St/AA submicrocapsules in the aqueous phase.

With the increase of [AA], the mean particle size and zeta potential of St/AA submicrocapsules were greatly decreased due to anionic AA and polyacrylic blocks in micelles and submicrocapsules, respectively. However, at over [AA]/[St] = 0.33, the mean particle size of St/AA submicrocapsules was increased, which was ascribed to the particle agglomeration in the aqueous phase due to the increased hydrophilic swollen layer of the particles and the resulting reduction in surface area and zeta potential. The increase in the applied amount of surfactant assures the reduction in size of micelles and resulting St/AA submicrocapsules. At higher [SDS] of over 1.0 × 10^−3^ mol, however, the formation of the weakened swollen layer, causing particle aggregation and a decrease in zeta potential, was suspected. Mean particle size and zeta potential of St/AA submicrocapsules were at the optimum condition at an initiator concentration of 2.0 × 10^−3^ mol/mol of monomer. Droplet nucleation was not effectively activated at a lower initiator concentration. At a higher initiator concentration, particle aggregation was manifested by virtue of the reduction in the portion of polyacrylic blocks in the swollen layer around the submicrocapsules.

From the above consideration, the optimum condition for miniemulsion copolymerization of St and AA was determined as follows; [AA]/[St] = 0.33, [SDS] = 1.0 × 10^−3^ mol, and [AIBN] = 2.0 × 10^−3^ mol/mol of monomer. Long-term colloidal stability was also evaluated for the optimum condition of St/AA submicrocapsules by measuring the optical transmission and the photon backscattering profiles of the aqueous dispersion. It is shown that backscattering was the prevalent signal and there were almost no variations in backscattering intensity, which means that St/AA submicrocapsules prepared at the optimum condition have superior colloidal stability without aggregation and sedimentation. These electrically stable St/AA submicrocapsules can be applied to various fields such as a targeted and/or sustained release drug delivery system, emulsion templating of 3D objects, additives for polymeric resins, highly dispersive fiber finishing agent, and so on.

## Figures and Tables

**Table 1:** Experimental conditions for miniemulsion copolymerization of St and AA.

Phase	Ingredients	Condition
Water phase	Distilled water	80 mL
	SDS	0.6~1.4 × 10^−3^ mol
Oil phase	Phytoncide oil	10 g
	DVB	250 mg
	*n*-Hexadecane	450 mg
	Monomer	0.172 mol
	(feed ratio [AA]/[St])	(0.2~1.0)
Homogenization	Rate	19,000 rpm
	Duration	10 min
